# Nuclear thymidylate synthase expression, p53 expression and 5FU response in colorectal carcinoma

**DOI:** 10.1054/bjoc.2001.2175

**Published:** 2001-12

**Authors:** N A C S Wong, L Brett, M Stewart, A Leitch, D B Longley, M G Dunlop, P G Johnston, A M Lessells, D I Jodrell

**Affiliations:** Department of Pathology, University of Edinburgh Medical School, Teviot Place, Edinburgh, EH8 9AG; ICRF Medical Oncology Unit, Department of Oncology, University of Edinburgh; MRC Human Genetics Unit, Western General Hospital, Edinburgh, EH4 2XU; Department of Oncology, The Queen's University of Belfast, Belfast City Hospital Tower, Belfast, BT9 7AB

**Keywords:** thymidylate synthase, colorectal carcinoma, immunohistochemistry, 5-fluorouracil, p53 protein, metastases

## Abstract

Thymidylate synthase (TS) is a key enzyme in DNA synthesis and is inhibited by metabolites of the chemotherapeutic agent 5-fluorouracil (5FU). Nuclear expression of TS in human tissue in vivo has not been characterised and its clinicopathological correlates in malignancy are unknown. 52 cases of primary colorectal carcinoma (CRC) and 24 cases of matched metastatic carcinoma were studied immunohistochemically using the monoclonal antibody TS106. The degree of nuclear TS immunostaining correlated closely with levels of TS mRNA expression amongst 10 CRCs studied. Strong nuclear immunostaining was seen in normal basal crypt colonocytes and germinal centre cells, and in a varying proportion of adenocarcinoma cells. Amongst the primary carcinomas, higher TS nuclear expression was associated with prominent extracellular mucin production and right-sided location. Higher TS nuclear expression also showed a significant association with poorer response to protracted venous infusional 5FU therapy. There was no clear association between TS nuclear expression and Ki67 or p53 expression assessed immunohistochemically. There was a strong positive correlation between TS nuclear expression in primary and metastatic CRC but the latter generally showed higher expression than matched primary tumour tissue. These findings confirm the nuclear expression of TS protein in human cells in vivo and provide new insight into how such expression may relate to the behaviour of CRCs. © 2001 Cancer Research Campaign http://www.bjcancer.com


British Journal of Cancer (2001) **85**(12), 1937–1943
© 2001 Cancer Research Campaign
doi: 10.1054/ bjoc.2001.2175, available online at http://www.idealibrary.com on IDEAL

http://www.bjcancer.com

### Nuclear thymidylate synthase expression, p53 expression and 5FU response in colorectal carcinoma

**NACS Wong[1], L Brett[1], M Stewart[2], A Leitch[3], DB Longley[4], MG Dunlop[3], PG Johnston[4], AM Lessells[1] and DI Jodrell[2]**

[1]Department of Pathology, University of Edinburgh Medical School, Teviot Place, Edinburgh EH8 9AG; [2]ICRF Medical Oncology Unit, Department of Oncology, University of Edinburgh, and [3]MRC Human Genetics Unit, Western General Hospital, Edinburgh EH4 2XU; [4]Department of Oncology, The Queen's University of Belfast, Belfast City Hospital Tower, Belfast BT9 7AB

**Summary** Thymidylate synthase (TS) is a key enzyme in DNA synthesis and is inhibited by metabolites of the chemotherapeutic agent 5-fluorouracil (5FU). Nuclear expression of TS in human tissue in vivo has not been characterised and its clinicopathological correlates in malignancy are unknown. 52 cases of primary colorectal carcinoma (CRC) and 24 cases of matched metastatic carcinoma were studied immunohistochemically using the monoclonal antibody TS106. The degree of nuclear TS immunostaining correlated closely with levels of TS mRNA expression amongst 10 CRCs studied. Strong nuclear immunostaining was seen in normal basal crypt colonocytes and germinal centre cells, and in a varying proportion of adenocarcinoma cells. Amongst the primary carcinomas, higher TS nuclear expression was associated with prominent extracellular mucin production and right-sided location. Higher TS nuclear expression also showed a significant association with poorer response to protracted venous infusional 5FU therapy. There was no clear association between TS nuclear expression and Ki67 or p53 expression assessed immunohistochemically. There was a strong positive correlation between TS nuclear expression in primary and metastatic CRC but the latter generally showed higher expression than matched primary tumour tissue. These findings confirm the nuclear expression of TS protein in human cells in vivo and provide new insight into how such expression may relate to the behaviour of CRCs. © 2001 Cancer Research Campaign http://www.bjcancer.com

**Keywords**: thymidylate synthase; colorectal carcinoma; immunohistochemistry; 5-fluorouracil; p53 protein; metastases

Thymidylate synthase (TS) is the rate-limiting enzyme in the production of thymine nucleotides, methylating deoxyuridine monophosphate (dUMP) to form deoxythymidine monophosphate (Peters et al, 1995). Inhibition of the enzyme is one of the main modes of action of the chemotherapeutic agent, 5-fluorouracil (5FU) (Thomas and Zalcberg, 1998). The drug is metabolised to form fluorodeoxyuridine monophosphate (FdUMP) which binds to TS, in competition with dUMP, to form a complex that deactivates the enzyme (Thomas and Zalcberg, 1998). The relation between TS and 5FU has led to a wealth of research regarding the enzyme's role in pre-existing and inducible resistance to the chemotherapeutic drug. With few exceptions (Findlay et al, 1997), existing data suggest increased tumour expression of TS protein predicts for a poorer response to 5FU in advanced colorectal carcinoma (CRC) (Peters et al, 1994; Johnston et al, 1995; Leichmann et al, 1995; Aschele et al, 1999; Paradiso et al, 2000). However, there are still unresolved issues regarding TS expression in CRC and its relation to 5FU therapy.

Firstly, most previous studies of TS protein expression and 5FU response had only analysed primary tumour tissue (Johnston et al, 1995; Findlay et al, 1997; Paradiso et al, 2000) whereas it is metastatic tumour that is targeted by 5FU therapy in patients with advanced CRC. We are aware of only two previous studies comparing TS expression between primary and metastatic tumour tissue (Peters et al, 1994; Aschele et al, 2000) and the two studies have produced conflicting results.

*Received 13 June 2001*
*Revised 17 September 2001*
*Accepted 20 September 2001*

*Correspondence to:* NACS Wong

Previous studies of TS protein expression in CRC in vivo have assessed such expression with either catalytic assays, FdUMP binding assays and/or ELISA analyses (Peters et al, 1995). However, one criticism of all these techniques is the inability to exclude TS expression of non-carcinoma cells, e.g. fibroblasts and inflammatory cells, in the samples analysed. Immunohistochemical assessment of TS protein expression provides an obvious method to address this problem. Until now, studies using this technique have only assessed cytoplasmic expression of the enzyme (Johnston et al, 1994, 1995; Suzuki et al, 1998; Yamachika et al, 1998; Paradiso et al, 2000). Despite the fact that nuclear staining has also been recorded (Johnston et al, 1991), we are unaware of any work into the clinicopathological significance of nuclear expression of TS in vivo. The presence of TS in the nucleus is of particular interest in light of the recent demonstration that TS protein and p53 mRNA form a biologically active complex and speculation that this complex may be localised within the nucleolus (Chu et al, 1999). There has also been interest in associations between TS and p53 on a more clinical level. At least two previous studies have shown that p53 protein expression may be used to supplement tumour TS protein expression as a predictor of response to 5FU therapy (Lenz et al, 1998a; Paradiso et al, 2000). Whether there is any relation between nuclear TS protein expression and p53 protein expression in vivo and whether the two may supplement one another in predicting for response of advanced CRC to 5FU therapy are unknown.

The aims of the following study were, therefore: (1) to confirm and characterise the nature of nuclear expression of TS in human

Data from this study were presented at the 179th Meeting of the Pathological Society of Great Britain and Ireland, held at Ninewells Hospital & Medical School, Dundee, UK.

tissue in vivo, concentrating on the colorectum; (2) to assess whether TS nuclear expression has any relation to clinicopathological features of CRC; (3) to study the relation between primary and metastatic tumour nuclear expression of TS protein; (4) to assess the relationship between TS nuclear protein expression and p53 protein expression in vivo; and (5) to study how these expressions relate to response to 5FU therapy for advanced CRC.

### MATERIALS AND METHODS

### Cases

The 52 patients studied had consented to participate in a study of protracted venous infusional 5-fluorouracil (PVI-5FU) for metastatic CRC (Jodrell et al, 2001). None of these patients had previously received any chemotherapeutic agents. The dose of 5FU that was used was 300 mg $m^{-2}$ $day^{-1}$ for 26 weeks and the patients had all started their chemotherapy between August 1995 and July 1997. This provided a median follow-up of 22.2 months (range: 16.8–28.5 months). Response to chemotherapy was defined, according to standard UICC guidelines, as complete response (CR), partial response (PR), no change (NC) or progressive disease (PD) (Hayward et al, 1977). Survival data was also collected for all 52 patients. Tissue blocks from the primary carcinoma and, where available, metastatic carcinoma (excluding lymph node metastases), of each patient were retrieved from the archival files of the Departments of Pathology at the University of Edinburgh Medical School, the Western General Hospital in Edinburgh, St John's Hospital in Livingston and the Royal Victoria Hospital in Kirkcaldy. Haematoxylin and eosin-stained sections of each primary tumour were reviewed by a gastrointestinal histopathologist (NW) to ensure uniformity in the assessment of the following pathological features: Dukes' stage, differentiation – based on guidelines released by the Royal College of Pathologists (Quirke and Williams, 1998) – and extracellular mucin production (abundant production was defined as extracellular mucin occupying > 50% of the tumour). Right-sided tumours were defined as those arising in the caecum, ascending colon or transverse colon, whereas left-sided tumours were defined as those arising in the descending colon, sigmoid colon or rectum.

### Immunohistochemistry

A number of 4 µm thick sections were cut from the tissue blocks, dewaxed and rehydrated. For TS immunostaining, antigen retrieval was carried out using Vector antigen retrieval fluid (Vector Laboratories, Peterborough, UK) and microwaving at full power for 10 min. Sections were pre-treated with 3% hydrogen peroxide for 10 min followed, after the antigen retrieval, by an avidin-blocking reagent (10% egg white; Sigma, Poole, UK) for 30 min and then the TS106 antibody to TS (from the laboratory of PGJ) at a dilution of 1:200 for 60 min. Visualisation was achieved by incubation with a 1:200 dilution of goat anti-mouse antibody (Vector Laboratories) for 30 min, followed by a streptavidin–biotin peroxidase complex (Dako, Ely, UK) for 30 min and finally diaminobenzidine (0.5 mg $m^{-1}$) for 5 min. For p53 immunostaining, the same protocol was used as for TS except 0.1 M citrate buffer (pH6) was used instead of Vector antigen retrieval fluid, and the primary antibody used was the DO-7 antibody to p53 (Novocastra, Newcastle, UK) at a dilution of 1:75. For Ki67 immunostaining, the same protocol was used as for p53 except the primary antibody used was the MM1 antibody to Ki67 (Novocastra) at a dilution of 1:100. Mayer's haematoxylin was used to counterstain the TS, p53 and Ki67 immunostained sections. A CRC found to express high nuclear levels of TS protein during preliminary studies was used as a positive control for TS immunohistochemistry. A CRC known to show p53 mutation and p53 protein overexpression was used as a positive control for p53 immunohistochemistry. A normal tonsil was used as a positive control for Ki67 immunohistochemistry. When present adjacent to tumour, lymphoid germinal centre cells and normal basal crypt colonocytes served as internal positive controls for both TS and Ki67 immunostaining. Finally, negative controls (i.e. lacking primary antibody) were included in each run of immunohistochemistry.

### TS mRNA analysis

RNA was isolated, as previously described (Gilmore et al, 1998), from tissue blocks of 10 of the 52 primary CRCs. Genomic DNA was removed from 2 µg of each RNA sample by digestion with DNase I, amplification grade (Gibco-BRL, Paisley, UK) according to the manufacturer's instructions. The RNA was then reverse transcribed using random hexamers as previously described (Horikoshi et al, 1992). Real-time quantitative polymerase chain reaction (PCR) amplification was performed using target-specific, doubly labelled fluorogenic probes with the ABI Prism 7700 Sequence Detection System (PE Applied Biosystems, Foster City, USA). TS primers and probe were selected with Primer Express software version 1.0 (PE Applied Biosystems). The TS probe was labelled with 6-carboxy-fluorescein (5′-end) and 6-carboxy-tetramethylrhodamine (3′-end). The specificity of the chosen sequence was confirmed by conducting a BlastN search (GenBank). The expression of the glyceraldehyde-3-phosphate dehydrogenase (GAPDH) gene was used as an internal standard using Pre-Developed TaqMan Assay Reagents for this gene (PE Applied Biosystems). The reactions were performed on an ABI Prism 7700 Sequence Detector using the standard PCR conditions recommended by PE Applied Biosystems for 60 cycles. During the PCR reaction, the reporter signal is normalised to an internal passive reference dye (ROX) to correct for any non-PCR-related fluctuations in fluorescence signal. The cycle number ($C_T$) at which the normalised fluorescent signal crosses a threshold set at 10 times the background fluorescence is related to the quantity of input DNA by an equation of the form: $C_T = -\,m\log(\text{input DNA}) + k$, where m and k are constants for a particular primer/probe set. $C_T$ values for the amplification of TS and GAPDH cDNAs were obtained for triplicate aliquots of each sample. Using serial dilutions of standard DNA samples, equations relating $C_T$ to the quantity of input DNA were established for TS and GAPDH, from which relative TS and GAPDH expression levels in each sample were determined. For TS, a DNA fragment containing the TS coding region was used as the reference standard. For GAPDH, cDNA prepared from the NCI-H630 colorectal cell line was used as the reference standard. That increased expression of GAPDH has been demonstrated in some human carcinomas, e.g. lung, breast and cervix (Revillion et al, 2000), suggests it may be suboptimal for use as a housekeeper gene for the study of these cancers. However, we are unaware of any data showing GAPDH expression to be up-regulated in CRC. Further, such up-regulation is associated with wide intertumoural variation of GAPDH mRNA levels (Revillion et al, 2000) whereas there was no obvious variation in GAPDH expression amongst the CRCs we studied.

 *© 2001 Cancer Research Campaign*

### Scoring

All TS-stained sections were assessed blindly and independently by 2 histopathologists (NW and AML). Both the percentage of positively stained nuclei and a histoscore were calculated for each case. Calculation of the histoscore (range of 0–300) – based on that used in the immunohistochemical assessment of oestrogen receptor expression (McCarty et al, 1985) – is explained in the legend for Figure 1. The percentage of positively stained nuclei was assessed semi-quantitatively by low to medium power histological examination (5–10 × objective) of all the tumour present on a section; percentages were rounded off to the nearest 5%. Assessment of the intensity of nuclear staining was made against an illustration showing examples of weak, moderate and strong intensity staining (Figure 1). The final TS histoscore used for each case was the mean of the 2 observers' individual scores. p53 immunostaining was scored by recording the percentage of nuclei showing strong positive staining from counting 500 carcinoma cells and then allocating each case to one of the following groups: I (< 10%); II (10 – 49%); III (50 – 89%); IV (90 – 100%). Ki67 immunostaining was scored by recording the percentage of nuclei showing positive staining from counting 500 carcinoma cells.

### Reproducibility and statistical analyses

All 76 TS-stained sections were re-scored by one of the observers (NW) to enable a calculation of intraobserver reproducibility. To assess the degree of variation of TS nuclear expression within any one tumour, five of the 52 primary carcinomas were selected at random and three additional tumour blocks retrieved for each of the five cases. Sections from these blocks were cut, stained with the TS antibody and scored. Kappa values were calculated to express intra- and interobserver reproducibilities using the computer software package MedCalc v5.0 (MedCalc Software, Mariakerke, Belgium). Mann–Whitney U tests and Spearman rank correlation tests were performed using the computer software package SPSS v7.5.1 for Windows 95 (SPSS Inc, USA). Both 2-tailed Fisher's exact tests and $\chi^2$ tests were performed using the computer software package Epi Info v6.04b (Centers for Disease Control & Prevention, Atlanta, USA). The relation of TS histoscore to patient survival was studied using standard Kaplan–Meier and logrank test analyses.

### RESULTS

### Clinicopathological details

Clinicopathological details of the primary tumours of the 52 patients (median age 60 years, range 33–77 years) are shown in Table 1. Non-nodal metastatic tissue was available for study from 24 patients: 13 liver metastases; seven omental/peritoneal metastases; and four ovarian metastases.

### TS immunohistochemistry

Immunohistochemical staining with TS antibody showed strong nuclear positivity in the germinal centres of secondary lymphoid

**Table 1** Clinicopathological features of 52 patients and their relation to TS histoscore

| Clinicopathological feature | Subgroups (no of patients) | Mean TS histoscore | *P* value[a] |
|---|---|---|---|
| Sex | Male (34) | 70.0 | 0.39 |
| | Female (18) | 72.5 | |
| Site | Right (20) | 95.0 | 0.08 |
| | Left (32) | 66.3 | |
| Dukes' stage | B (11) | 72.5 | 0.85[b] |
| | C (28) | 68.8 | |
| | D (13) | 70.0 | 0.96[b] |
| Differentiation | Well (2) | 65.0 | 0.75[c] |
| | Moderate (43) | 68.8 | |
| | Poor (7) | 90.0 | 0.15[c] |
| Abundant extra cellular mucin | Yes (10) | 90.0 | 0.03 |
| | No (42) | 67.5 | |

[a]Mann–Whitney U test. [b]Dukes' B compared with Dukes' C tumours, and Dukes' C compared with Dukes' D tumours. [c]Well differentiated compared with moderately differentiated tumours, and moderately differentiated compared with poorly differentiated tumours.

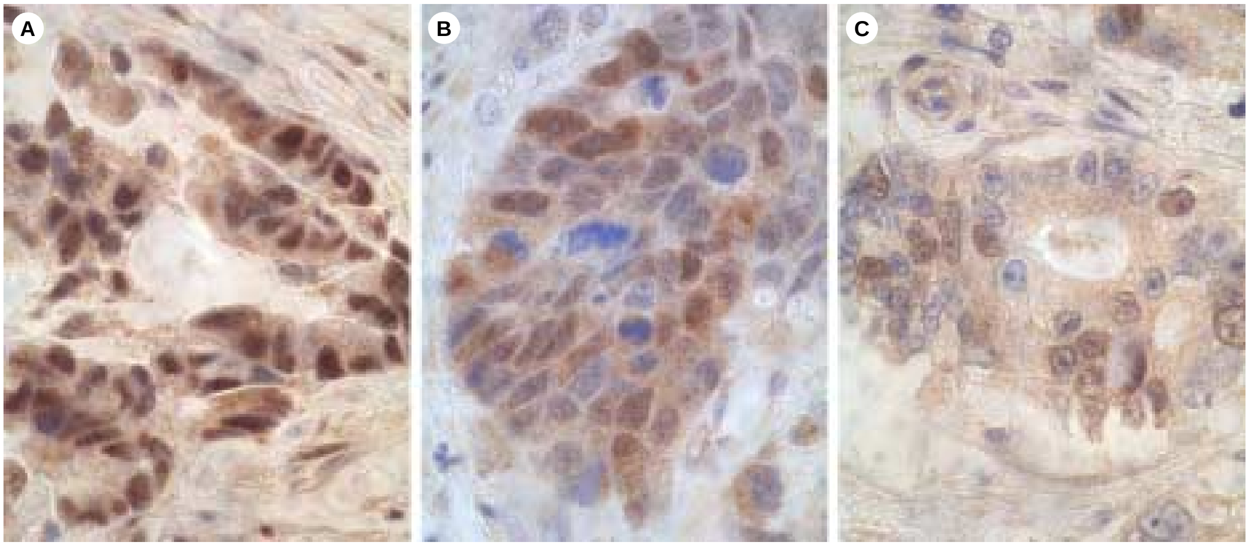

**Figure 1** Examples of (**A**) strong, (**B**) moderate, and (**C**) weak intensity TS nuclear staining in colorectal carcinomas (all × 100 obj). TS histoscore = (percentage of tumour cells showing strong intensity staining × 3) + (percentage showing moderate intensity staining × 2) + (percentage showing weak intensity staining × 1). For example, a tumour with 20% of its cells showing strong intensity staining, 30% showing moderate intensity staining, 30% showing weak intensity staining and 20% showing no staining would be given a histoscore of (20 × 3) + (30 × 2) + (30 × 1) = 150

*© 2001 Cancer Research Campaign*

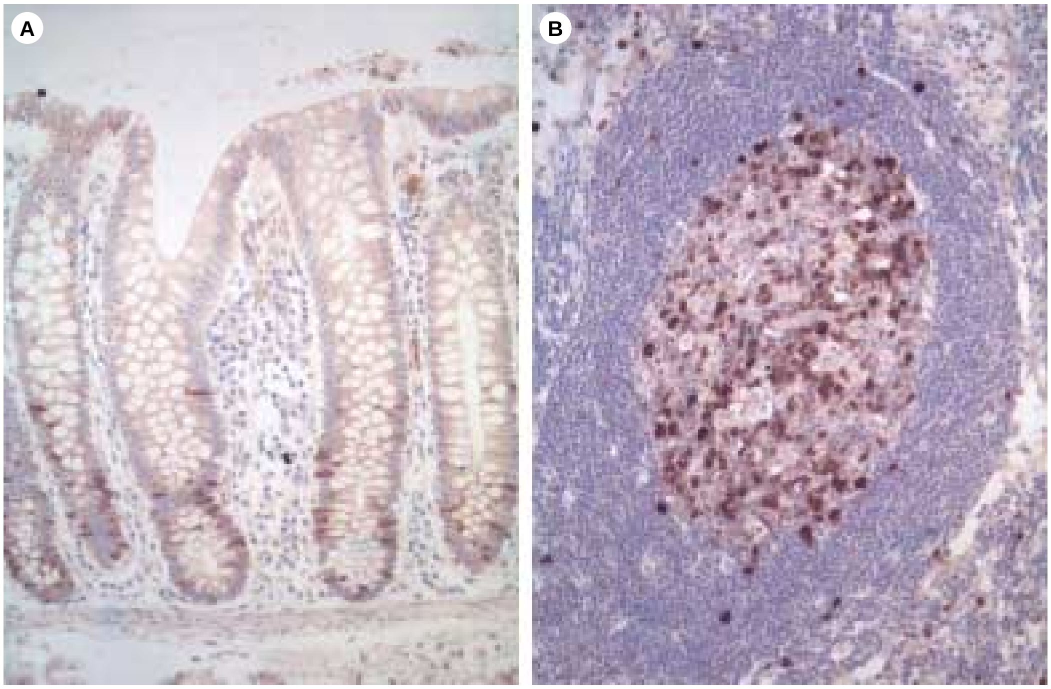

**Figure 2** TS immunostaining (all × 20 obj): (**A**) normal colonic mucosa showing nuclear staining confined to the lower third crypt colonocytes; (**B**) lymphoid follicle showing nuclear staining restricted primarily to germinal centre cells follicles and in the basal third epithelial cells of normal colonic crypts (Figure 2). When present in CRCs, strong and moderate intensity nuclear staining was often associated with prominent cytoplasmic staining (Figure 1). A formal correlative study of nuclear versus cytoplasmic expression of TS was not considered valid, as our immunostaining protocol had been specifically designed to optimise nuclear and not cytoplasmic staining.

### TS histoscores

There was good intraobserver (Spearman rank correlation coefficient 0.83, kappa coefficient 0.62) and interobserver reproducibility (Spearman rank correlation coefficient 0.75, kappa coefficient 0.52) in the calculation of TS histoscores. For any one of the five tumours used to test intratumoural variation of TS nuclear expression, there was a maximum difference in TS histoscore of 15 amongst sections from four tumour blocks. TS histoscore showed a significant, positive correlation (Spearman rank correlation coefficient 0.71, $P$ = 0.022) with TS mRNA expression measured from 10 primary CRCs (Figure 3).

The distribution of TS histoscores amongst the 52 primary carcinomas is shown in Figure 4; the median TS histoscore was 70. The relation of TS nuclear expression to different clinicopathological features of the 52 cases of primary CRC is shown in Table 1. Of all the features studied, only the presence of abundant extracellular mucin showed a statistically significant association with TS nuclear expression.

There were no differences in TS histoscores between liver and other sites of metastases (data not shown). Amongst the 24 pairs of primary and metastatic tumour, the latter – median TS histoscore 105 – showed significantly greater nuclear TS expression than primary tumour – median TS histoscore 70 – (Mann–Whitney U test, $P$ = 0.01). The relationship between primary and metastatic tumour nuclear TS expression is shown in Figure 5. There was a significant positive correlation between the TS histoscores of primary and matched metastatic tumour (Spearman rank correlation coefficient 0.62, $P$ = 0.001). Further, nine of the 13 (69%) primary tumours with below median (< 70) TS histoscores had matched metastatic tumour TS histoscores of below the median (< 105), and nine of the 11 (82%) primary tumours with above median TS histoscores had matched metastatic tumour TS histoscores of above the median ($\chi^2$ test, $P$ = 0.04).

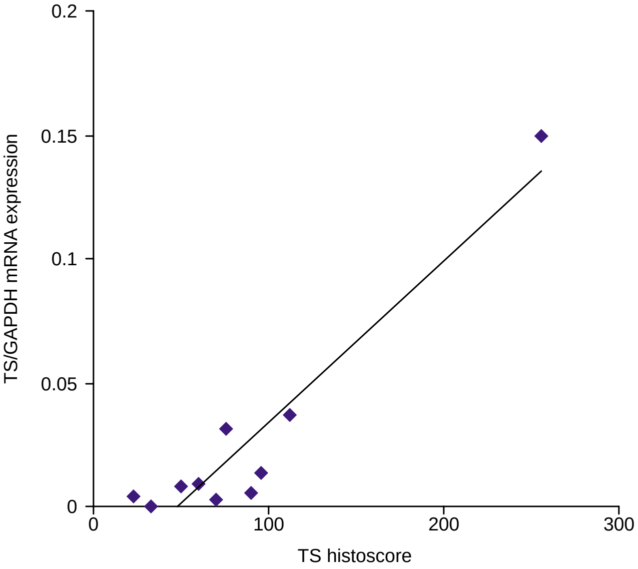

**Figure 3** Relationship between TS histoscores and TS mRNA expression measured by real-time quantitative PCR from 10 primary colorectal carcinomas. A line of best fit is also shown

 *© 2001 Cancer Research Campaign*

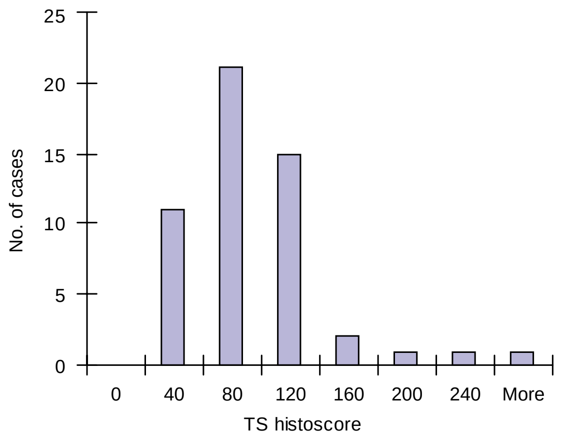

**Figure 4** Histogram showing distribution of TS histoscores amongst 52 primary colorectal carcinomas

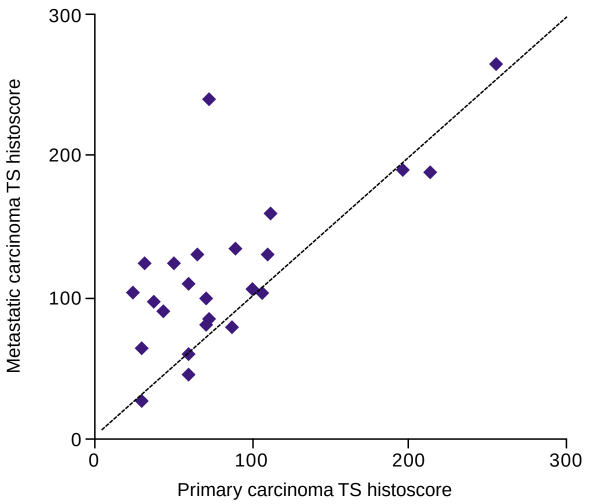

**Figure 5** Scatterplot of TS histoscores of 24 matched cases of primary and metastatic colorectal carcinoma. A line of unity is also shown

### Response to 5FU therapy and survival

Of the 52 patients, 16 (30.8%) showed CR or PR, 18 (34.6%) showed NC and 18 (34.6%) showed PD. 12 (75%) of the 16 responding patients had primary tumour TS histoscores of less than the group median (i.e. 70) while four (25%) patients had primary tumour histoscores of greater than 70. By contrast, 16 (44%) of the 36 non-responding patients had primary tumour TS histoscores of less than the group median while 20 (56%) patients had primary tumour histoscores of greater than 70 ($\chi^2$ test, $P$ = 0.04). The proportion of patients with primary tumour TS histoscores of greater than 70 increased with decreasing response to 5FU: 25% of CR/PR patients, 44% of NC patients and 67% of PD patients ($\chi^2$ test, $P$ = 0.05). Finally, the median TS histoscore (60.0) of carcinomas that demonstrated a response to 5FU (CR and PR) was less than that (92.5) of carcinomas showing progression (PD) though this trend just failed to reach statistical significance at the 95% level (Mann–Whitney U test, $P$ = 0.06). Assessing TS expression using only the percentage of positively stained primary tumour nuclei failed to show an obvious difference between responding and non-responding patients (data not shown). None of the clinicopathological features of the primary tumours shown in Table 1 were significantly associated with 5FU response (data not shown). The proportion of patients with metastatic tumour TS histoscores greater than the group median (i.e. 105) increased with decreasing response to 5FU: 29% of CR/PR patients, 43% of NC patients and 80% of PD patients. However, this trend failed to reach statistical significance ($\chi^2$ test, $P$ = 0.09). Survival data was available for all 52 patients. There were no significant associations between TS histoscore and cause-specific or overall survival (data not shown).

### p53 and Ki67 data

There were 15 primary carcinomas in p53 group I, 11 in group II, 11 in group III and 15 in group IV. There was no obvious relation between TS histoscore and p53 group amongst either primary or metastatic carcinomas (data not shown). Comparing primary carcinomas showing or not showing p53 overexpression (defined as >10% nuclear staining, i.e. groups II–IV) with those showing TS histoscores greater or less than the group median also failed to demonstrate any significant association ($\chi^2$ test, $P$ = 0.53).

The median Ki67-labelling index amongst the 52 primary carcinomas was 45% (range 10–91%). There was no obvious relation between TS histoscore and Ki67-labelling index (data not shown). There was no significant association between Ki67-labelling index and response to 5FU (data not shown).

### DISCUSSION

This study has demonstrated that TS is expressed within the nuclei of normal colonocytes, normal lymphoid cells and colorectal adenocarcinoma cells. The prominence of nuclear expression in the base of normal colonic crypts and in the germinal centres of lymphoid follicles is in keeping with these sites representing areas of active cell proliferation and, hence, DNA synthesis. Current literature on the subcellular localisation of TS in human cells in vivo is lacking. However, previous studies have demonstrated the presence of the enzyme in the nuclei of rat hepatoma cells using immunogold electron microscopy and autoradiography (Samsonoff et al, 1997), and along the nuclear periphery of yeasts using immunofluorescence analysis (Poon and Storms, 1994). The significance of this subcellular localisation is uncertain but, as previously mentioned, TS protein and p53 mRNA form a complex which is speculated to have a nucleolar location (Chu et al, 1999). TS protein is also known to bind to other mRNAs, e.g. c-myc mRNA (Chu et al, 1996), but whether and how nuclear TS regulates expression of their corresponding proteins remains to be determined.

Our histoscore technique was shown to be reproducible and its ability to quantify nuclear expression of TS supported by good correlation with TS mRNA expression measured using real-time quantitative PCR. The importance of considering intensity of TS immunostaining is demonstrated by our failure to show an association between 5FU response and TS expression assessed by calculating only the percentage of stained nuclei. Interestingly, the study of metastatic CRC that failed to demonstrate a correlation between response to 5FU or Tomudex (another TS inhibitor) and TS protein expression had quantified the latter by assessing only the percentage of tumour area showing immunogold staining (Findlay et al, 1997). The validity of our histoscore technique is further supported by the fact that the positively skewed distribution of TS histoscores seen amongst our carcinomas (Figure 4) is similar to the distributions (our analysis of the authors' data) of TS expression

© 2001 Cancer Research Campaign measured amongst primary CRCs using FdUMP binding (Larsson et al, 1998; Sanguedolce et al, 1998) or enzyme activity assays (Edler et al, 1997). The disproportionate number of carcinomas lying in the upper tails of these distributions is of particular interest as they may represent a distinct subpopulation with regards to TS protein expression. All of our 3 carcinomas (patients' ages: 33, 52 and 77 years) with TS histoscores of greater than 190 were right-sided and showed prominent extracellular mucin, features that are characteristic of microsatellite instability (MSI)-associated CRCs (Toft and Arends, 1998). Therefore, although the effect of MSI on 5FU response remains controversial (Carethers et al, 1999; El Saleh et al, 2000; Hemminki et al, 2000), it would be interesting to formally study the relations between TS expression, MSI and 5FU response in CRC in vivo.

Our finding that secondary CRC tissue expresses greater nuclear TS protein than primary tissue differs from the results of the 2 existing studies on this matter. Peters and colleagues found no consistent differences in TS expression between primary and secondary CRC tissue (Peters et al, 1994). However, these investigators assessed TS expressions by catalytic and FdUMP binding assays rather than immunohistochemistry, and only 14 pairs of tissue were studied compared with 24 in our study. Using an immunohistochemical method on 27 pairs of tissue, Aschele and colleagues reported a lack of correlation of cytoplasmic TS protein expression between primary and metastatic CRC tissue, and overall higher expression in the former (Aschele et al, 2000). While it is uncertain why our findings are diametrically opposite to those of Aschele and colleagues, this discrepancy may relate to differential expression of TS protein in the nucleus compared with the cytoplasm, as is discussed below. Clonal selection is a recognised component of the process of metastasis (De Both et al, 1997). As an explanation for our findings, clones of increased nuclear TS-expressing cells may be selected for when CRCs metastasise. The survival advantage gained by such selection is unclear but may relate to TS's putative role as a regulator of several oncogenic proteins, such as c-myc. Further, TS expression has recently been shown, in CRCs, to correlate with expression of vascular endothelial growth factor (van Triest et al, 2000), a compound important to the process of metastasis (Bikfalvi, 1995). In keeping with a positive correlation between primary and metastatic tissue TS nuclear expression, a higher TS histoscore in CRC metastases appeared to associate with poorer response to 5FU therapy. The fact that this association did not reach statistical significance might merely be due to a smaller sample size.

The association between higher TS nuclear protein expression and poorer response to 5FU therapy is in keeping with several previous studies of advanced CRC where TS cytoplasmic protein expression was measured by immunohistochemical or biochemical techniques or TS mRNA expression was measured by reverse transcriptase PCR (Peters et al, 1994; Johnston et al, 1995; Lenz et al, 1998b; Paradiso et al, 2000). In support of published data (Brett et al, 1996), none of the clinicopathological details shown in Table 1 individually predicted for 5FU response. It therefore seems unlikely that TS nuclear expression is only indirectly associated with 5FU response via a relation with a recognised clinicopathological feature of CRC. Another possible confounding factor relates to previous work demonstrating an association between cell proliferation indices and 5FU response in a variety of tumour types (Ravaioli et al, 1998; Zhang et al, 1998). In view of the role of TS in DNA synthesis, it may be speculated that the TS histoscore predicts for 5FU response amongst primary CRCs only by acting as a cell proliferation index. This, however, is unlikely in view of the lack of direct association between Ki67-labelling index and 5FU response and the poor correlation between the former and TS histoscore. This lack of correlation in malignant tissue contrasts against the prominence of TS nuclear expression at sites of cell proliferation in normal tissue, as noted above. It may therefore be suggested that, in malignancy, the expression of nuclear TS protein is dysregulated and no longer tied closely to the level of DNA synthesis and proliferative activity.

There is increasing evidence of interactions between the TS and p53 systems. As previously mentioned, TS protein has been shown to bind to p53 mRNA, thereby reducing expression of p53 protein (Chu et al, 1999). Trying to predict, from these findings, the association between TS and p53 proteins in vivo is, however, complicated by observations that wild-type p53 protein can, in turn, inhibit TS promoter activity (Lee et al, 1997). Indeed, this second interaction better explains the in vivo observations that p53 gene mutation and protein overexpression (an accepted though not absolute surrogate marker of mutant protein expression) associates with increased TS mRNA (Lenz et al, 1998b) and cytoplasmic protein (Lenz et al, 1998a; Paradiso et al, 2000) expression amongst CRCs. We, however, were unable to demonstrate any association between increased nuclear TS expression and p53 overexpression. Our sample size (52 patients) was larger than those used in most previous studies, e.g. 36 (Lenz et al, 1998b) and 45 (Lenz et al, 1998a). Further, we and, for example, Lenz and colleagues used the same monoclonal antibody clones (TS106 for TS and DO-7 for p53) and the same definition of p53 overexpression (greater than 10% positively stained nuclei). It may therefore be speculated that p53 overexpression has different effects on cytoplasmic TS expression compared with nuclear expression. Some evidence for such differential regulation of TS expression derives from Maley and colleagues' studies on rat hepatoma cells. Cells exposed to increasing concentrations of fluorodeoxyuridine show increased TS protein levels (Rhee et al, 1990) but this change is predominantly seen in the cytoplasm as opposed to the nucleus (Samsonoff et al, 1997). Further work will be required to clarify the reasons for and the mechanisms underlying this differential expression of TS, if not just to help understand the interaction between p53 and the enzyme.

In summary, we have confirmed the presence of nuclear expression of TS protein in human colorectal tissue in vivo and investigated associations of such expression with different clinicopathological features of CRC. The relatively small sample of CRCs studied suggests our data and conclusions should be viewed as being preliminary. However, they do point toward further studies particularly relating to the use of nuclear TS expression to predict for 5FU response. It would be informative to compare nuclear against cytoplasmic TS protein expression and TS mRNA expression as predictors of such response and survival amongst a large cohort of CRC patients. Finally, whether measuring thymidine phosphorylase (Salonga et al, 2000; van Triest et al, 2000) and dihydropyrimidine dehydrogenase (Salonga et al, 2000) expression improves the ability of TS nuclear expression to predict for 5FU response also deserves investigation.

### ACKNOWLEDGEMENTS

This project was funded by the Imperial Cancer Research Fund and a grant from the Western General Hospital Research & Development Fund.

 *© 2001 Cancer Research Campaign*

**REFERENCES**

Aschele C, Debernardis D, Casazza S, Antonelli G, Tunesi G, Baldo C, Lionetto R, Maley F and Sobrero A (1999) Immunohistochemical quantitation of thymidylate synthase expression in colorectal cancer metastases predicts for clinical outcome to fluorouracil-based chemotherapy. *J Clin Oncol* **17**: 1760–1770

Aschele C, Debernardis D, Tunesi G, Maley F and Sobrero A (2000) Thymidylate synthase protein expression in primary colorectal cancer compared with the corresponding distant metastases and relationship with the clinical response to 5-fluorouracil. *Clin Cancer Res* **6**: 4797–4802

Bikfalvi A (1995) Significance of angiogenesis in tumour progression and metastasis. *Eur J Cancer* **31A**: 1101–1104

Brett MC, Pickard M, Green B, Howel-Evans A, Smith D, Kinsella A and Poston G (1996) p53 protein overexpression and response to biomodulated 5-fluorouracil chemotherapy in patients with advanced colorectal cancer. *Eur J Surg Oncol* **22**: 181–185

Carethers JM, Chauhan DP, Fink D, Nebel S, Bresalier RS, Howell SB and Boland CR (1999) Mismatch repair proficiency and in vitro response to 5-fluorouracil. *Gastroenterology* **117**: 123–131

Chu E, Cogliate T, Copur SM, Borre A, Voeller DM, Allegra CJ and Segal S (1996) Identification of in vivo target RNA sequences bound by thymidylate synthase. *Nucl Acid Res* **24**: 3222–3228

Chu E, Copur SM, Chen TM, Khleif S, Voeller DM, Mizunuma N, Patel M, Maley GF, Maley F and Allegra CJ (1999) Thymidylate synthase protein and p53 mRNA form an in vivo ribonucleoprotein complex. *Mol Cell Biol* **19**: 1582–1594

De Both NJ, Vermey M, Groen N, Dinjens WN and Bosman FT (1997) Clonal growth of colorectal-carcinoma cell lines transplanted to nude mice. *Int J Cancer* **72**: 1137–1141

Edler D, Blomgren H, Allegra CJ, Johnston PG, Lagerstedt U, Magnusson I and Ragnhammar P (1997) Immunohistochemical determination of thymidylate synthase in colorectal cancer – methodogical studies. *Eur J Cancer* **33**: 2278–2281

Elsaleh H, Joseph D, Grieu F, Zeps N, Spry N and Iacopetta B (2000) Association of tumour site and sex with survival benefit from adjuvant chemotherapy in colorectal cancer. *Lancet* **355**: 1745–1750

Findlay MPN, Cunningham D, Morgan G, Clinton S, Hardcastle A and Aherne GW (1997) Lack of correlation between thymidylate synthase levels in primary colorectal tumours and subsequent response to chemotherapy. *Br J Cancer* **75**: 903–909

Gilmore PM, Church SW, Danenberg KD, Danenberg PV, Salonga D, Park JM and Johnston PG (1998) The development and optimization of a quantitative RT-PCR technique from formalin-fixed and paraffin-embedded (FFPE) tissues, using the thymidylate synthase gene as a target [abstract]. *Proc Am Soc Clin Oncol* **17**: 2159

Hayward JL, Carbone PP, Heuson J-C, Kumaoka S, Segaloff A and Rubens RD (1977) Assessment of response to therapy in advanced breast cancer. *Eur J Cancer* **13**: 89–94

Hemminki A, Mecklin JP, Jarvinen H, Aaltonen LA and Joensuu H (2000) Microsatellite instability is a favorable prognostic indicator in patients with colorectal cancer receiving chemotherapy. *Gastroenterology* **119**: 921–928

Horikoshi T, Danenberg KD, Stadlbauer TH, Volkenandt M, Shea LC, Aigner K, Gustavsson B, Leichman L, Frosing R and Ray M (1992) Quantitation of thymidylate synthase, dihydrofolate reductase, and DT-diaphorase gene expression in human tumors using the polymerase chain reaction. *Cancer Res* **52**: 108–116

Jodrell DI, Stewart M, Aird R, Knowles G, Bowman A, Wall L, Cummings J and McLean C (2001) 5-fluorouracil steady state pharmacokinetics and outcome in patients receiving protracted venous infusion for advanced colorectal cancer. *Br J Cancer* **84**: 600–603

Johnston PG, Liang C-M, Henry S, Chabner BA and Allegra CJ (1991) Production and characterisation of monoclonal antibodies that localise human thymidylate synthase in the cytoplasm of human cells and tissue. *Cancer Res* **51**: 6668–6676

Johnston PG, Fisher ER, Rockette HE, Fisher B, Wolmark N, Drake JC, Chabner BA and Allegra CJ (1994) The role of thymidylate synthase expression in prognosis and outcome in adjuvant chemotherapy in patients with rectal cancer. *J Clin Oncol* **12**: 2640–2647

Johnston PG, Lenz H-J, Leichman CG, Danenberg KD, Allegra CJ, Danenberg PV and Leichman L (1995) Thymidylate synthase gene and protein expression correlate and are associated with response to 5-fluorouracil in human colorectal and gastric tumors. *Cancer Res* **55**: 1407–1412

Larsson P-A, Carlsson G, Larsson L and Gustavsson B (1998) Determination of relative thymidylate synthase (TS) gene expression in tumour specimens and normal colon mucosa – relation to TS protein levels. *GI Cancer* **2**: 293–299

Lee Y, Chen Y, Chang L-S and Johnson LF (1997) Inhibition of mouse thymidylate synthase promotor activity by the wild-type p53 tumor suppressor protein. *Exp Cell Res* **234**: 270–276

Leichman L, Lenz HJ, Leichman CG, Groshen S, Danenberg K, Baranda J, Spears CP, Boswell W, Silberman H, Ortega A, Stain S, Beart R and Danenberg P (1995) Quantitation of intratumoral thymidylate synthase expression predicts for resistance to protracted infusion of 5-fluorouracil and weekly leucovorin in disseminated colorectal cancers: preliminary report from an ongoing trial. *Eur J Cancer* **31A**: 1306–1310

Lenz H-J, Danenberg KD, Leichman CG, Florentine B, Johnston PG, Groshen S, Zhou L, Xiong YP, Danenberg PV and Leichman LP (1998a) p53 and thymidylate synthase expression in untreated stage II colon cancer: association with recurrence, survival and site. *Clin Cancer Res* **4**: 1227–1234

Lenz H-J, Hayashi K, Salonga D, Danenberg KD, Danenberg PV, Metzger R, Banerjee D, Bertino JR, Groshen S, Leichman LP and Leichman CG (1998b) p53 point mutation and thymidylate synthase messenger RNA levels in disseminated colorectal cancer: an analysis of response and survival. *Clin Cancer Res* **4**: 1243–1250

McCarty KS Jr., Miller LS, Cox EB, Konrath J and McCarty KS Sr. (1985) Estrogen receptor analyses. Correlation of biochemical and immunohistochemical methods using monoclonal antibodies. *Arch Pathol Lab Med* **109**: 716–721

Paradiso A, Simone G, Petroni S, Leone B, Vallejo C, Lacava J, Romero A, Machiavelli M, De Lena M, Allegra CJ and Johnston PG (2000) Thymidylate synthase and p53 primary tumour expression as predictive factors for advanced colorectal cancer patients. *Br J Cancer* **82**: 560–567

Peters GJ, van der Wilt CL, van Groeningen CJ, Smid K, Meijer S and Pinedo HM (1994) Thymidylate synthase inhibition after administration of fluorouracil with or without leucovorin in colon cancer patients: implications for treatment with fluorouracil. *J Clin Oncol* **12**: 2035–2042

Peters GJ, van der Wilt CL, van Triest B, Codacci-Pisanelli G, Johnston PG, van Groeningen CJ and Pinedo HM (1995) Thymidylate synthase and drug resistance. *Eur J Cancer* **31A**: 1299–1305

Poon P-P and Storms RK (1994) Thymidylate synthase is localized to the nuclear periphery in the yeast Saccharomyces cerevisiae. *J Biol Chem* **269**: 8341–8347

Quirke P and Williams GT (1998) Minimum dataset for colorectal cancer histopathology reports. London: The Royal College of Pathologists (UK)

Ravaioli A, Bagli L, Zucchini A and Monti F (1998) Prognosis and prediction of response in breast cancer: the current role of the main biological markers. *Cell Proliferation* **31**: 113–126

Revillion F, Pawlowski V, Hornez L and Peyrat JP (2000) Glyceraldehyde-3-phosphate dehydrogenase gene expression in human breast cancer. *Eur J Cancer* **36**: 1038–1042

Rhee MS, Balinska M, Bunni M, Priest DG, Maley GF, Maley F and Galivan J (1990) Role of substrate depletion in the inhibition of thymidylate biosynthesis by the dihydrofolate reductase inhibitor trimetrexate in cultured hepatoma cells. *Cancer Res* **50**: 3979–3984

Salonga D, Danenberg KD, Johnson M, Metzger R, Groshen S, Tsao-Wei DD, Lenz HJ, Leichman CG, Leichman L, Diasio RB and Danenberg PV (2000) Colorectal tumors responding to 5-fluorouracil have low gene expression levels of dihydropyrimidine dehydrogenase, thymidylate synthase, and thymidine phosphorylase. *Clin Cancer Res* **6**: 1322–1327

Samsonoff WA, Reston J, McKee M, O'Connor B, Galivan J, Maley G and Maley F (1997) Intracellular location of thymidylate synthase and its state of phosphorylation. *J Biol Chem* **272**: 13281–13285

Sanguedolce R, Vultaggio G, Sanguedolce F, Modica G, Li Volsi F, Diana G, Guereio G, Bellanca L and Rausa L (1998) The role of thymidylate synthase levels in the prognosis of the treatment of patients with colorectal cancer. *Anticancer Res* **18**: 1515–1520

Suzuki M, Okabe H, Tsukagoshi S, Kawai T, Fukushima M and Sato I (1998) Immunohistochemical investigation of thymidylate synthase in cervical cancer. *Oncology* **55**: 564–568

Thomas DM and Zaleberg JR (1998) 5-fluorouracil: a pharmacological paradigm in the use of cytotoxics. *Clin Exp Pharmacol Physiol* **25**: 887–895

Toft NJ and Arends MJ (1998) DNA mismatch repair and colorectal cancer. *J Pathol* **185**: 123–129

van Triest B, Pinedo HM, Blaauwgeers JL, van Diest PJ, Schoenmakers PS, Voorn DA, Smid K, Hoekman K, Hoitsma HF and Peters GJ (2000) Prognostic role of thymidylate synthase, thymidine phosphorylase/platelet-derived endothelial cell growth factor, and proliferation markers in colorectal cancer. *Clin Cancer Res* **6**: 1063–1072

Yamachika T, Nakanishi H, Inada K-I, Tsukamoto T, Kato T, Fukushima M, Inoue M and Tatematsu M (1998) A new prognostic factor for colorectal carcinoma, thymidylate synthase and its therapeutic significance. *Cancer* **82**: 70–77

Zhang XD, Coventry BJ, Jamieson GG and Gill PG (1998) The utility of the proliferative index in pretreatment biopsy specimens of esophageal squamous cell carcinoma. *Disease of the Esophagus* **11**: 215–220

*© 2001 Cancer Research Campaign*

## References

[PDF_00584] Aschele C., Debernardis D., Casazza S., Antonelli G., Tunesi G., Baldo C., Lionetto R., Maley F., Sobrero A. (1999). Immunohistochemical quantitation of thymidylate synthase expression in colorectal cancer metastases predicts for clinical outcome to fluorouracil-based chemotherapy.. J Clin Oncol.

[PDF_00588] Aschele C., Debernardis D., Tunesi G., Maley F., Sobrero A. (2000). Thymidylate synthase protein expression in primary colorectal cancer compared with the corresponding distant metastases and relationship with the clinical response to 5-fluorouracil.. Clin Cancer Res.

[PDF_00592] Bikfalvi A. (1995). Significance of angiogenesis in tumour progression and metastasis.. Eur J Cancer.

[PDF_00594] Brett M. C., Pickard M., Green B., Howel-Evans A., Smith D., Kinsella A., Poston G. (1996). p53 protein overexpression and response to biomodulated 5-fluorouracil chemotherapy in patients with advanced colorectal cancer.. Eur J Surg Oncol.

[PDF_00598] Carethers J. M., Chauhan D. P., Fink D., Nebel S., Bresalier R. S., Howell S. B., Boland C. R. (1999). Mismatch repair proficiency and in vitro response to 5-fluorouracil.. Gastroenterology.

[PDF_00601] Chu E., Cogliati T., Copur S. M., Borre A., Voeller D. M., Allegra C. J., Segal S. (1996). Identification of in vivo target RNA sequences bound by thymidylate synthase.. Nucleic Acids Res.

[PDF_00604] Chu E., Copur S. M., Ju J., Chen T. M., Khleif S., Voeller D. M., Mizunuma N., Patel M., Maley G. F., Maley F. (1999). Thymidylate synthase protein and p53 mRNA form an in vivo ribonucleoprotein complex.. Mol Cell Biol.

[PDF_00607] De Both N. J., Vermey M., Groen N., Dinjens W. N., Bosman F. T. (1997). Clonal growth of colorectal-carcinoma cell lines transplanted to nude mice.. Int J Cancer.

[PDF_00610] Edler D., Blomgren H., Allegra C. J., Johnston P. G., Lagerstedt U., Magnusson I., Ragnhammar P. (1997). Immunohistochemical determination of thymidylate synthase in colorectal cancer--methodological studies.. Eur J Cancer.

[PDF_00613] Elsaleh H., Joseph D., Grieu F., Zeps N., Spry N., Iacopetta B. (2000). Association of tumour site and sex with survival benefit from adjuvant chemotherapy in colorectal cancer.. Lancet.

[PDF_00616] Findlay M. P., Cunningham D., Morgan G., Clinton S., Hardcastle A., Aherne G. W. (1997). Lack of correlation between thymidylate synthase levels in primary colorectal tumours and subsequent response to chemotherapy.. Br J Cancer.

[PDF_00628] Hemminki A., Mecklin J. P., Järvinen H., Aaltonen L. A., Joensuu H. (2000). Microsatellite instability is a favorable prognostic indicator in patients with colorectal cancer receiving chemotherapy.. Gastroenterology.

[PDF_00631] Horikoshi T., Danenberg K. D., Stadlbauer T. H., Volkenandt M., Shea L. C., Aigner K., Gustavsson B., Leichman L., Frösing R., Ray M. (1992). Quantitation of thymidylate synthase, dihydrofolate reductase, and DT-diaphorase gene expression in human tumors using the polymerase chain reaction.. Cancer Res.

[PDF_00635] Jodrell D. I., Stewart M., Aird R., Knowles G., Bowman A., Wall L., McLean C. (2001). 5-fluorouracil steady state pharmacokinetics and outcome in patients receiving protracted venous infusion for advanced colorectal cancer.. Br J Cancer.

[PDF_00642] Johnston P. G., Fisher E. R., Rockette H. E., Fisher B., Wolmark N., Drake J. C., Chabner B. A., Allegra C. J. (1994). The role of thymidylate synthase expression in prognosis and outcome of adjuvant chemotherapy in patients with rectal cancer.. J Clin Oncol.

[PDF_00646] Johnston P. G., Lenz H. J., Leichman C. G., Danenberg K. D., Allegra C. J., Danenberg P. V., Leichman L. (1995). Thymidylate synthase gene and protein expression correlate and are associated with response to 5-fluorouracil in human colorectal and gastric tumors.. Cancer Res.

[PDF_00639] Johnston P. G., Liang C. M., Henry S., Chabner B. A., Allegra C. J. (1991). Production and characterization of monoclonal antibodies that localize human thymidylate synthase in the cytoplasm of human cells and tissue.. Cancer Res.

[PDF_00653] Lee Y., Chen Y., Chang L. S., Johnson L. F. (1997). Inhibition of mouse thymidylate synthase promoter activity by the wild-type p53 tumor suppressor protein.. Exp Cell Res.

[PDF_00656] Leichman L., Lenz H. J., Leichman C. G., Groshen S., Danenberg K., Baranda J., Spears C. P., Boswell W., Silberman H., Ortega A. (1995). Quantitation of intratumoral thymidylate synthase expression predicts for resistance to protracted infusion of 5-fluorouracil and weekly leucovorin in disseminated colorectal cancers: preliminary report from an ongoing trial.. Eur J Cancer.

[PDF_00662] Lenz H. J., Danenberg K. D., Leichman C. G., Florentine B., Johnston P. G., Groshen S., Zhou L., Xiong Y. P., Danenberg P. V., Leichman L. P. (1998). p53 and thymidylate synthase expression in untreated stage II colon cancer: associations with recurrence, survival, and site.. Clin Cancer Res.

[PDF_00666] Lenz H. J., Hayashi K., Salonga D., Danenberg K. D., Danenberg P. V., Metzger R., Banerjee D., Bertino J. R., Groshen S., Leichman L. P. (1998). p53 point mutations and thymidylate synthase messenger RNA levels in disseminated colorectal cancer: an analysis of response and survival.. Clin Cancer Res.

[PDF_00671] McCarty K. S., Miller L. S., Cox E. B., Konrath J., McCarty K. S. (1985). Estrogen receptor analyses. Correlation of biochemical and immunohistochemical methods using monoclonal antireceptor antibodies.. Arch Pathol Lab Med.

[PDF_00675] Paradiso A., Simone G., Petroni S., Leone B., Vallejo C., Lacava J., Romero A., Machiavelli M., De Lena M., Allegra C. J. (2000). Thymidilate synthase and p53 primary tumour expression as predictive factors for advanced colorectal cancer patients.. Br J Cancer.

[PDF_00679] Peters G. J., van der Wilt C. L., van Groeningen C. J., Smid K., Meijer S., Pinedo H. M. (1994). Thymidylate synthase inhibition after administration of fluorouracil with or without leucovorin in colon cancer patients: implications for treatment with fluorouracil.. J Clin Oncol.

[PDF_00683] Peters G. J., van der Wilt C. L., van Triest B., Codacci-Pisanelli G., Johnston P. G., van Groeningen C. J., Pinedo H. M. (1995). Thymidylate synthase and drug resistance.. Eur J Cancer.

[PDF_00686] Poon P. P., Storms R. K. (1994). Thymidylate synthase is localized to the nuclear periphery in the yeast Saccharomyces cerevisiae.. J Biol Chem.

[PDF_00688] Ravaioli A., Bagli L., Zucchini A., Monti F. (1998). Prognosis and prediction of response in breast cancer: the current role of the main biological markers.. Cell Prolif.

[PDF_00696] Rhee M. S., Balinska M., Bunni M., Priest D. G., Maley G. F., Maley F., Galivan J. (1990). Role of substrate depletion in the inhibition of thymidylate biosynthesis by the dihydrofolate reductase inhibitor trimetrexate in cultured hepatoma cells.. Cancer Res.

[PDF_00693] Révillion F., Pawlowski V., Hornez L., Peyrat J. P. (2000). Glyceraldehyde-3-phosphate dehydrogenase gene expression in human breast cancer.. Eur J Cancer.

[PDF_00700] Salonga D., Danenberg K. D., Johnson M., Metzger R., Groshen S., Tsao-Wei D. D., Lenz H. J., Leichman C. G., Leichman L., Diasio R. B. (2000). Colorectal tumors responding to 5-fluorouracil have low gene expression levels of dihydropyrimidine dehydrogenase, thymidylate synthase, and thymidine phosphorylase.. Clin Cancer Res.

[PDF_00705] Samsonoff W. A., Reston J., McKee M., O'Connor B., Galivan J., Maley G., Maley F. (1997). Intracellular location of thymidylate synthase and its state of phosphorylation.. J Biol Chem.

[PDF_00708] Sanguedolce R., Vultaggio G., Sanguedolce F., Modica G., Li Volsi F., Diana G., Guereio G., Bellanca L., Rausa L. (1998). The role of thymidylate synthase levels in the prognosis and the treatment of patients with colorectal cancer.. Anticancer Res.

[PDF_00712] Suzuki M., Okabe H., Tsukagoshi S., Kawai T., Fukushima M., Sato I. (1998). Immunohistochemical investigation of thymidylate synthase in cervical cancer.. Oncology.

[PDF_00715] Thomas D. M., Zalcberg J. R. (1998). 5-fluorouracil: a pharmacological paradigm in the use of cytotoxics.. Clin Exp Pharmacol Physiol.

[PDF_00717] Toft N. J., Arends M. J. (1998). DNA mismatch repair and colorectal cancer.. J Pathol.

[PDF_00724] Yamachika T., Nakanishi H., Inada K., Tsukamoto T., Kato T., Fukushima M., Inoue M., Tatematsu M. (1998). A new prognostic factor for colorectal carcinoma, thymidylate synthase, and its therapeutic significance.. Cancer.

[PDF_00727] Zhang X. D., Coventry B. J., Jamieson G. G., Gill P. G. (1998). The utility of the proliferative index in pretreatment biopsy specimens of esophageal squamous cell carcinoma.. Dis Esophagus.

[PDF_00719] van Triest B., Pinedo H. M., Blaauwgeers J. L., van Diest P. J., Schoenmakers P. S., Voorn D. A., Smid K., Hoekman K., Hoitsma H. F., Peters G. J. (2000). Prognostic role of thymidylate synthase, thymidine phosphorylase/platelet-derived endothelial cell growth factor, and proliferation markers in colorectal cancer.. Clin Cancer Res.

